# Early transcriptomic changes at the skin interface during Powassan virus transmission by *Ixodes scapularis* ticks

**DOI:** 10.3389/fimmu.2024.1511132

**Published:** 2025-01-13

**Authors:** Dakota N. Paine, Meghan Hermance, Saravanan Thangamani

**Affiliations:** ^1^ Department of Microbiology and Immunology, State University of New York Upstate Medical University, Syracuse, NY, United States; ^2^ State University of New York Center for Vector-Borne Diseases, State University of New York Upstate Medical University, Syracuse, NY, United States; ^3^ Department of Microbiology and Immunology, University of South Alabama, Mobile, AL, United States; ^4^ Upstate Global Health Institute, State University of New York Upstate Medical University, Syracuse, NY, United States

**Keywords:** Powassan virus, Ixodes, immunomodulation, transmission, arbovirus

## Abstract

**Introduction:**

Powassan virus (POWV), a vector-borne pathogen transmitted by *Ixodes* ticks in North America, is the causative agent of Powassan encephalitis. As obligate hematophagous organisms, ticks transmit pathogens like POWV at the tick bite site, specifically during the initial stages of feeding. Tick feeding and salivary factors modulate the host’s immunological responses, facilitating blood feeding and pathogen transmission. However, the mechanisms of immunomodulation during POWV transmission remain inadequately understood. In this study, we investigated the global cutaneous transcriptomic changes associated with tick bites during POWV transmission.

**Methods:**

We collected skin biopsies from the tick attachment sites at 1, 3, and 6 h after feeding by POWV-infected and uninfected ticks, followed by RNA sequencing of these samples. Differentially expressed genes were analyzed for pathway enrichment using gene ontology and pathway enrichment analyses.

**Results:**

Our findings reveal that tick feeding alone significantly impacts the skin transcriptome within the first 1 to 3 h of tick attachment. Although early POWV transmission induces minimal changes in the local environment, a pronounced shift toward a proinflammatory state is observed 6 h after tick attachment, characterized by neutrophil recruitment and interleukin signaling.

**Discussion:**

These transcriptomic data elucidate the dynamic changes at the tick bite site, transitioning from changes that assist blood meal acquisition to a proinflammatory phase that may facilitate viral dissemination.

## 1 Introduction

The prevalence and geographical range of ticks are expanding globally, particularly in North America, where *Ixodes scapularis* has extended its range due to climate change, which increasingly favors the tick’s preferred habitats (1). This expansion poses significant public health risks, as *I. scapularis* is a primary vector for several pathogens, including bacteria such as *Borrelia burgdorferi* and *Anaplasma phagocytophilum*, as well as viral pathogens like Powassan virus (POWV) and deer tick virus (DTV) (2). POWV, first isolated in 1958 from the brain of a young boy who succumbed to Powassan encephalitis (3), can cause severe neurological sequelae in survivors, including hemiplegia, muscle wasting, and memory deficits (4, 5). Despite the severity of the disease, there is currently no antiviral treatment or U.S. Food and Drug Administration (FDA)-approved vaccine. However, recent advances in DNA-based, as well as live-attenuated vaccine platforms, show promise in eliciting a strong protective immune response against POWV. The latter utilizes the historically successful yellow fever virus 17D backbone, which has seen licensed success when used for Japanese encephalitis virus and dengue virus (6, 7).

Arthropod saliva plays a crucial role in transmitting vector-borne pathogens (8). During blood feeding, ticks insert their mouthparts into the host’s skin and secrete saliva into the bite site. Tick saliva contains a variety of bioactive components that facilitate feeding by enabling the tick to remain attached, acquire a complete blood meal, and evade the host’s immune defenses (9–11). Pathogens have evolved to exploit these salivary components, enhancing their transmission to the host in a process known as saliva-assisted transmission (12–14).

Mouse studies have demonstrated that POWV transmission can occur in as little as 15 min of tick feeding, a significantly shorter duration compared to that of other pathogens transmitted by ticks, such as *Borrelia burgdorferi*, which typically requires 48–72 h of tick feeding but has been observed in as early as 24 h after tick attachment, for transmission to occur (15–17). Understanding the early host responses to POWV transmission is crucial, as these early time points are characterized by robust fluctuations in the gene expression of various cytokines and chemokines (18). Previous studies have shown that co-injection with tick salivary gland extract and POWV results in enhanced neuroinvasion in BALB/c mice compared to POWV injection alone (14). Additionally, feeding by POWV-infected *I. scapularis* induces a significant influx of neutrophils and macrophages to the bite site within the first few hours, indicating strong immunological signaling during POWV transmission (19). However, the specific immunomodulatory events during the initial hours of POWV transmission from tick to host still need to be understood.

**Figure 1:**
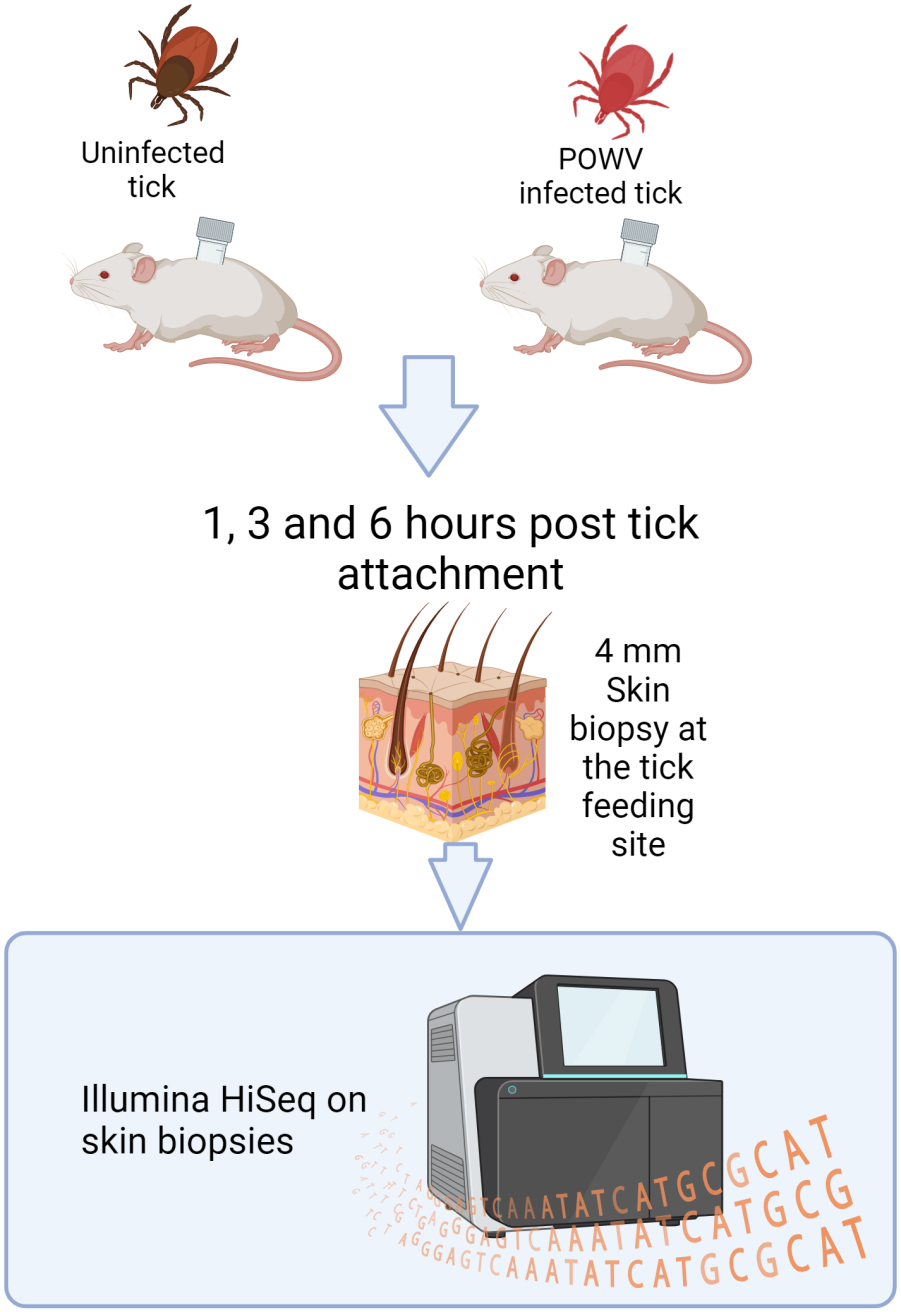
Design of an *I. scapularis* feeding study with downstream RNA-seq analysis. POWV-infected ticks or uninfected ticks were allowed to feed on mice for 1, 3, or 6 h. After feeding, the ticks were removed, and the bite sites were biopsied and processed for Illumina HiSeq sequencing.

This study explores the global transcriptomic changes at the tick bite site during the first hours of POWV transmission. We employed a POWV-infected *I. scapularis* feeding model to investigate the differential cutaneous gene expression profiles at 1, 3, and 6 h after tick attachment (**Figure 1**). Our findings highlight a proinflammatory response characterized by chemokine-mediated neutrophil recruitment and degranulation. This provides new insights into the host-pathogen interactions during tick-borne POWV transmission.

## 2 Materials and methods

### 2.1 Ethics statement

All experiments involving mice and infected ticks were conducted in arthropod containment level 3 facilities in strict accordance with an animal use protocol approved by the University of Texas Medical Branch (UTMB) Institutional Animal Care and Use Committee (IACUC; # 0907054).

### 2.2 Viral culture

African green monkey kidney cells (Vero E6) were acquired from American Type Culture Collection and maintained in Dulbecco’s modified Eagle’s medium (DMEM) supplemented with 10% fetal bovine serum and 1% antibiotic cocktail consisting of penicillin and streptomycin and incubated at 37°C with 5% CO_2_. The prototypic LB strain of POWV was acquired from the World Reference Center for Emerging Viruses and Arboviruses at the UTMB, which had been passaged seven times in suckling mice brains. The stock of the virus was then passaged six times in Vero E6 cells. Stock viral titers were determined via focus-forming assay as previously described (20). Next-generation sequencing revealed that the consensus nucleotide sequence of our POWV stock is 99.96% identical to the original POWV_LB isolated in Ontario (GenBank: NC_003687.1). There were three nucleotide changes between our POWV stock and the POWV LB isolate, leading to a single–amino acid change in the E protein (Q1436R), as well as one nucleotide gap that may have caused a nucleotide shift in the NS5 protein (W8255 to L or S).

### 2.3 Animals

Five-week-old female BALB/cj mice were acquired from The Jackson Laboratory (Bar Harbor, ME). Mice were allowed to acclimate to the local environment for 1 week before the start of experiments, at which point the mice were 6 weeks old.

### 2.4 Tick infection and infestation on mice

The mouse skin biopsies used in this study were generated as a part of another study that described tick salivary gland microRNA expression during POWV transmission (21). Briefly, uninfected adult *I. scapularis* were maintained in our lab within the UTMB arthropod containment level 2 facilities. Tick vials were stored inside an incubator at 22°C and 90%–93% humidity. The incubator photoperiod was set to a 16:8-h cycle. Adult, female *I. scapularis* were synchronously infected with POWV-LB strain as previously described (18). Control female ticks were mock-infected with media. After the infection, *I. scapularis* females were co-housed with uninfected male *I. scapularis* and stored inside a desiccator at 22°C to allow POWV replication. Mock-infected females were co-housed with male *I. scapularis* in the same manner. At 40 days after synchronous infection, the *I. scapularis* females were infested on mice.

Tick containment capsules were prepared as previously described (18). Two days before tick infestation, mice were anesthetized with isoflurane, and the backs of each mouse were shaved. One capsule was adhered to the dorsum of each mouse using Kamar adhesive (Kamar Inc., Steamboat Springs, CO). On the day of tick infestation, one *I. scapularis* female was placed inside each capsule and allowed to feed on the mouse for 1, 3, or 6 h. Three biological replicates were included for each of tick feeding time points described in this study. Time points were measured on the basis of the observed attachment time for each individual tick, for example, once embedding of the hypostome is visually observed, a timer was started for that individual tick. This was to ensure accurate feeding times across groups. At the experimental time points, mice were euthanized with CO_2_ inhalation according to IACUC protocols. The feeding tick was removed, and 4-mm skin biopsies were dissected from each mouse at the tick feeding site. Salivary glands from the removed tick were also dissected to check POWV infection status. Each skin biopsy and tick salivary glands were stored for RNA extraction in QIAzol lysis reagent (Qiagen). In the POWV-infected tick group, we confirmed the POWV-infection status prior to be included in this study (21). Three mice skin biopsies from each group and time points were used for RNA sequencing (RNA-seq) analysis described in this study.

### 2.5 RNA extractions, q-RT-PCR, and digital droplet PCR detection of POWV RNA

Four-millimeter skin biopsies and tick salivary glands, in QIAzol, were homogenized in QIAzol (Qiagen). Following homogenization, RNA extraction was performed using an RNeasy mini kit (Qiagen), yielding purification of total RNA. Viral infection in the skin and tick salivary glands were verified via digital droplet (ddPCR) and Quantitative Reverse transcription polymerase chain reaction (q-RT-PCR), respectively, using POWV primers (POWV-F: CCGAGCCAAAGTGAGGATGT and POWV-R: TCTTTTGCCGAGCTCCACTT) as previously described (22). For the salivary glands, the following cycling protocol was used: 10 min at 50°C; 1 min at 95°C; 10 s at 95°C followed by 30 s at 60°C for 45 cycles; and an 81-cycle (+0.5°C/cycle) 55°C–95°C melt curve.

For POWV RNA detection in the skin, given the small amount of viral RNA that was expected to be present in the skin at the time of feeding, we opted for ddPCR. To start, 15-µL reactions were prepared consisting of QIAcuity OneStep Advanced Probe Kit (QIAgen) materials and template RNA. On a QIAcuity, nucleic acids were amplified with the following cycling conditions: 40 min at 50°C, 2 min at 95°C, 40 cycles of 5 s at 95°C, and 30 s at 60°C. Images were then taken with an exposure time of 500 ms and a gain of 6 using the green channel. Thresholds were calculated by manually adjusting according to a negative control.

### 2.6 Library preparation with PolyA selection and Illumina sequencing

The RNA samples were quantified using Qubit 2.0 Fluorometer (Life Technologies, Carlsbad, CA, USA), and, then, the RNA integrity was verified using Agilent TapeStation 4200 (Agilent Technologies, Palo Alto, CA, USA).

RNA-seq libraries were prepared using the NEBNext Ultra II RNA Library Prep Kit for Illumina, based on the manufacturer’s instructions (New England Biolabs (NEB), Ipswich, MA, USA). Briefly, mRNAs were enriched with Oligo(dT) beads. Enriched mRNAs were fragmented for 15 min at 94°C. First-strand and second-strand Complementary deoxyribonucleic acid (cDNAs) were subsequently synthesized. cDNA fragments were end-repaired and adenylated at 3’ends, and universal adapters were ligated to cDNA fragments, followed by index addition and library enrichment by limited-cycle PCR. Sequencing libraries were validated on the Agilent TapeStation (Agilent Technologies, Palo Alto, CA, USA) and quantified by using Qubit 2.0 Fluorometer (Invitrogen, Carlsbad, CA) as well as by quantitative PCR (KAPA Biosystems, Wilmington, MA, USA). The sequencing libraries were clustered on flow cells. After clustering, the flow cells were loaded onto the Illumina HiSeq instrument (4000 or equivalent) according to the manufacturer’s instructions. The samples were sequenced using a 2 × 150-bp paired-end configuration. Image analysis and base calling were conducted by the HiSeq Control Software. Raw sequence data (.bcl files) generated from Illumina HiSeq was converted into fastq files and de-multiplexed using Illumina’s bcl2fastq 2.17 software. One mismatch was allowed for index sequence identification.

### 2.7 Data analysis

After investigating the quality of the raw data, sequence reads were trimmed to remove possible adapter sequences and nucleotides with poor quality using Trimmomatic v.0.36. The trimmed reads were then mapped to the Mus musculus reference genome available on ENSEMBL using the STAR aligner v.2.5.2b.

After the extraction of gene hit counts, the gene hit count table was used for downstream differential expression analysis. Using DESeq2, a comparison of gene expression between the groups of samples was performed. The Wald test was used to generate p-values and log2 fold changes. Genes with adjusted p-values <0.05 and absolute log2 fold changes >1 were called as differentially expressed genes (DEGs) for each comparison. Raw and analyzed data have been deposited to the Gene Expression Omnibus under accession number GSE282786. A gene ontology (GO) analysis was performed on the statistically significant set of genes by implementing the software GeneSCF. The gene ontology annotation (goa) human GO list was used to cluster the set of genes based on their biological process and determine their statistical significance. A principal component analysis (PCA) analysis was performed using the “plotPCA” function within the DESeq2 R package. The plot shows the samples in a 2D plane spanned by their first two principal components: infection status, tick feeding, or time, depending on the bioinformatic comparison. The top 500 genes, selected by highest row variance, were used to generate the plot. In the event where genes were identified in one group but not another, they were denoted as 1.0 log2 fold increase and treated as such.

## 3 Results

### 3.1 Transcriptomic changes at the skin interface during *I. scapularis* timed blood meal acquisition

Pathogen-free *I. scapularis* females were allowed to feed on naïve mice for 1, 3, and 6 h. Subsequent to feeding, skin biopsies from the tick feeding site were collected for RNA-seq and differential gene expression analysis following RNA extraction. Naïve skin from tick-free, uninfected mice was used as a control. The analysis revealed that 3,722, 3,210, and 3,905 genes were differentially regulated compared to tick-free control skin after 1, 3, and 6 h of feeding of uninfected ticks, respectively (**Table 1**). Additionally, POWV-infected *I. scapularis* females were allowed to feed on naïve mice for 1, 3, and 6 h, with their skin transcriptomic profiles compared to those of uninfected ticks at corresponding time points. The total number of DEGs identified in these POWV-infected versus uninfected tick feeding site comparisons were 1, 20, and 83 for 1, 3, and 6 h of feeding, respectively (**Table 1**).

**Table 1:** Total expressed genes in each comparison made for uninfected and POWV-infected *I. scapularis* feedings.

Comparison	Upregulated genes	Downregulated genes	Total significantly differentially expressed genes
naiveControl-vs-1HourUninfected	2,231	1,491	3,722
naiveControl-vs-3HourUninfected	1,819	1,391	3,210
naiveControl-vs-6HourUninfected	2,162	1,743	3,905
1HourUninfected-vs-1HourPOWV	0	1	1
3HourUninfected-vs-3HourPOWV	9	11	20
6HourUninfected-vs-6HourPOWV	80	3	83

#### 3.1.1 Naïve skin vs. skin from the site of uninfected *I. scapularis* fed for 1 h

**Figure 2:**
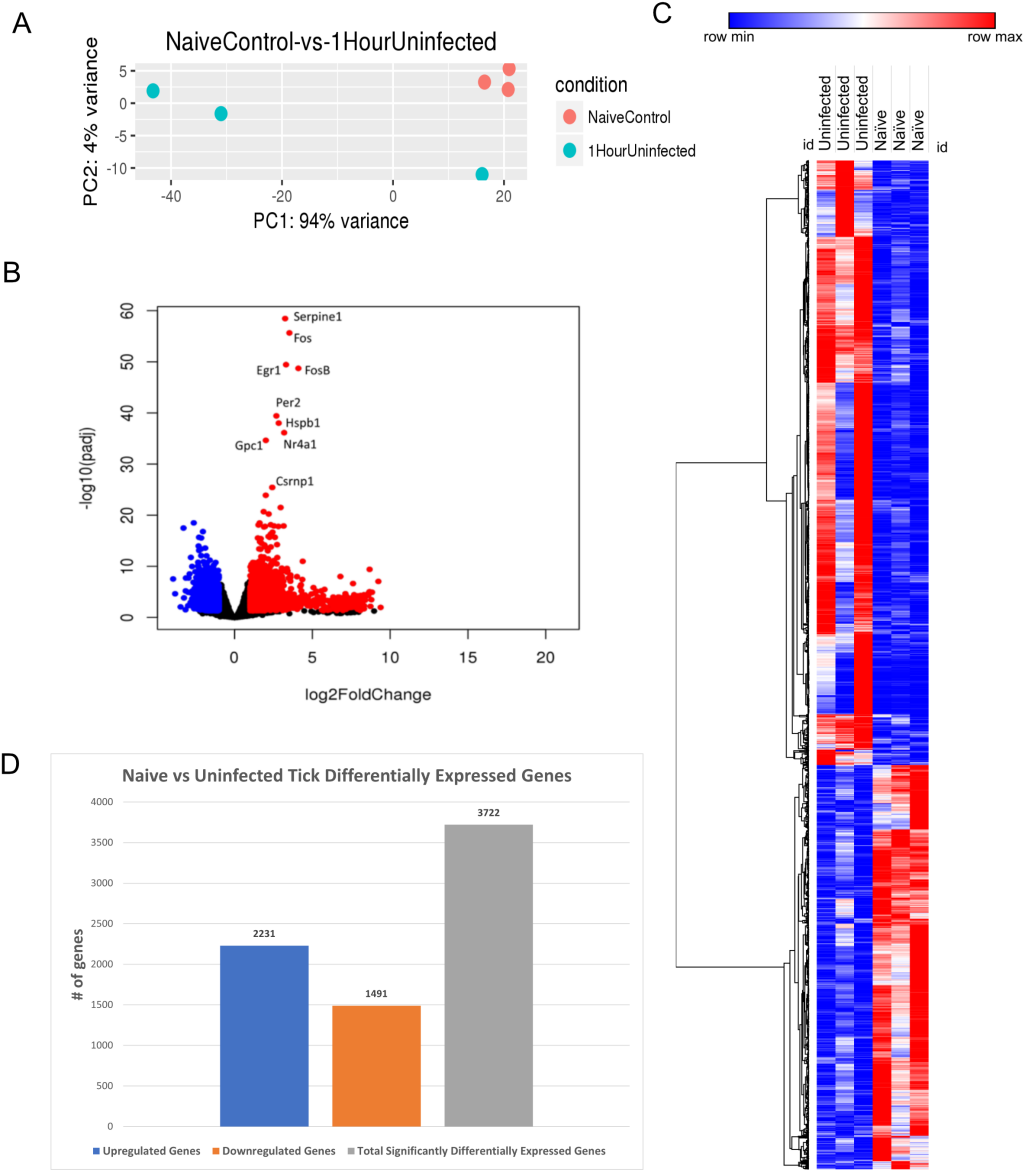
*I. scapularis*–free skin compared to 1-h uninfected *I. scapularis* feeding. **(A)** Principal component analysis (PCA) of transcriptional profiles when comparing 1-h uninfected tick feeding sites (blue) to naïve tick-free skin (orange). **(B)** Volcano plot outlining upregulated and downregulated genes at 1 h. The x-axis identifies the log2 fold change of every gene, and the y-axis identifies the log10 of its adjusted p-value. Only genes with a p-value <0.05 and a log2 fold change <−1 are identified as downregulated (blue). Only genes that have a p-value <0.05 and a log2 fold change >1 are identified as upregulated (red). **(C)** A bi-clustering heatmap was used to visualize the expression profile of all differentially expressed genes (DEGs), sorted by their adjusted p-value by plotting log2-transformed values in samples. **(D)** Total DEGs for uninfected tick feeding vs. naïve skin at 1 h.

We first analyzed the cutaneous transcriptomic changes induced by an uninfected tick feeding for 1 h. PCA was conducted to assess their component profiles (**Figure 2A**).

A volcano plot was generated to illustrate the global transcriptional changes across the groups. Genes with a p-value <0.05 and a log2 fold change <−1 were identified as downregulated (blue), whereas genes with a p-value <0.05 and a log2 fold change >1 were identified as upregulated (red) (**Figure 2B**). A total of 3,722 DEGs were identified and visualized in the volcano plot, with significantly upregulated outliers highlighted. Notably, *Serpine1*, *Fos*, *FosB*, *EGR1*, *HSPB1*, and *GPC1* were highly upregulated and statistically significant at this feeding time point. These genes are associated with functions such as wound healing, blood clotting, stress response, and cell proliferation, which are directly relevant to tick feeding. Additionally, several immune regulatory genes were differentially expressed during the 1-h feeding, including *MAPK* and its pathway components like *JUN*, *IL-6*, and early expression of *NFκB* components. These findings are consistent with earlier studies reporting similar results from *I. scapularis* feedings (23). A bi-clustering heatmap analysis was performed to examine the complete transcriptomic profile of each sample, with hierarchical sorting based on Pearson correlation (**Figure 2C**). The heatmap indicates slight differential regulation between the skin harvested from tick-free control mice and the 1-h fed uninfected tick feeding site. Although the genes represented in the heatmap exhibited modest changes, they generally showed increased expression compared to the naïve controls. The differential analysis revealed a total of 3,722 genes expressed in this study (**Figure 2D**).

The DEG data were further analyzed using the Reactome database, and pathway enrichment (PE) analysis was conducted for significant DEGs during the 1-h feeding. The analysis revealed impacted pathways, including keratinization, formation of the cornified envelope, and NGF-stimulated transcription and nuclear events, with the latter being specific to kinase and transcription factor activation (**Table 2**).

**Table 2:** Pathway enrichment of naïve *I. scapularis*–free skin vs. skin harvested after 1 h of uninfected *I. scapularis* feeding.

Pathway identifier	Pathway name	#Entities found	#Entities total	Entities ratio	Entities p-value	Entities FDR
R-HSA-6805567	Keratinization	112	226	0.014837	9.60E-10	2.01E-06
R-HSA-6809371	Formation of the cornified envelope	57	138	0.00906	0.001047	0.980202
R-HSA-9031628	NGF-stimulated transcription	26	56	0.003676	0.005486	0.980202
R-HSA-198725	Nuclear events (kinase and transcription factor activation)	32	80	0.005252	0.01724	0.980202
R-HSA-390522	Striated muscle contraction	18	40	0.002626	0.023957	0.980202
R-HSA-8939256	RUNX1 regulates transcription of genes involved in WNT signaling	6	9	5.91E-04	0.03503	0.980202

Only pathways with a p-value <0.05 are shown. The entire table is found in **Supplementary Table S1**.

#### 3.1.2 Naïve skin vs. skin from the site of uninfected *I. scapularis* fed for 3 h

**Figure 3:**
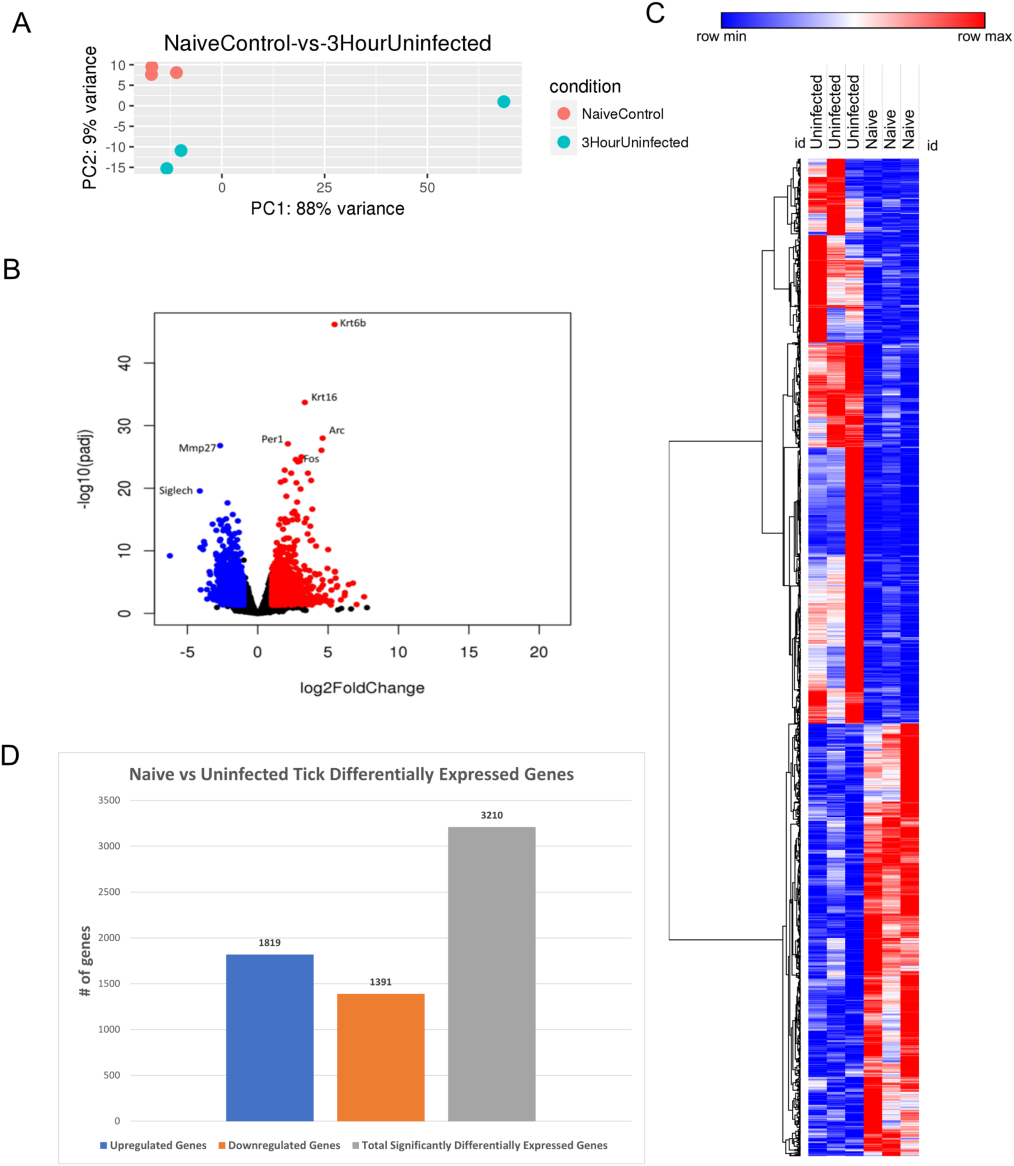
Differential expression of genes in *I. scapularis*–free control skin compared to 3-h uninfected *I. scapularis* feedings. **(A)** Principal component analysis (PCA) of transcriptional profiles when comparing 3-h uninfected feedings (blue) to naïve skin (orange). **(B)** Volcano plot outlining upregulated and downregulated genes at 3 h. The x-axis identifies the log2 fold change of every gene, and the y-axis specifies the log10 of its adjusted p-value. Only genes with a p-value <0.05 and a log2 fold change <−1 are identified as downregulated (blue). Only genes with a p-value <0.05 and a log2 fold change >1 are identified as upregulated (red). **(C)** A bi-clustering heatmap was used to visualize the expression profile of all differentially expressed genes (DEGs), sorted by their adjusted p-value by plotting log2-transformed values in samples. **(D)** Total DEGs for uninfected tick feeding vs. naïve skin at 3 h.

We next investigated the cutaneous transcriptomic changes occurring during 3 h of uninfected tick feeding. The PCA revealed clustering of individual samples for the tick-free control group, with some variance observed in the skin of the 3-h uninfected tick cohort (**Figure 3A**).

A volcano plot was used to analyze the most DEGs. The plot identified 3,210 DEGs, with 1,819 upregulated and 1,391 downregulated genes (**Figure 3B**). Upregulated genes involved in the keratin gene family, including *Krt6b* and *Krt16*, and downregulated metalloproteases such as *Mmp27* were observed. With both *Krt6b* and *Krt16* being upregulated, there could be potential mechanisms for wound healing, directly in response to tick blood meal acquisition. *Siglech*, a gene involved in cell-cell interactions and regulatory functions of the innate immune system through polysaccharide interactions, was also downregulated (24). Additionally, *Arc*, *Per1*, and *Fos* were upregulated, corresponding to functions such as cytoskeletal growth and sodium ion retention (25). *Fos* acts upstream of transcription factor AP-1, which regulates cell proliferation, differentiation, and transformation (26).

A bi-clustering heatmap of all significantly DEGs was generated as previously described (**Figure 3C**). The differences between the two groups were more pronounced than the 1-h uninfected feeding heatmap (**Figure 2C**). This summary heatmap shows the comparisons of each individual mouse for both groups: naïve/tick-free skin versus skin harvested from the 3-h uninfected tick feeding site. The tree to the left demonstrates the hierarchical clustering of the samples. The differential analysis revealed a total of 3,210 genes expressed in this study (**Figure 3D**). DEG data were input into the Reactome database, and PE analysis revealed the impacted pathways during the 3-h feeding (**Table 3**).

**Table 3:** Pathway enrichment of naïve *I. scapularis*–free skin vs. skin harvested after 3 h of uninfected *I. scapularis* feedings.

Pathway identifier	Pathway name	#Entities found	#Entities total	Entities ratio	Entities p-value	Entities FDR
R-HSA-9031628	NGF-stimulated transcription	23	56	0.003676	0.007192	0.973472
R-HSA-3656244	Defective B4GALT1 causes B4GALT1-CDG (CDG-2d)	6	9	5.91E-04	0.019633	0.973472
R-HSA-3656225	Defective CHST6 causes MCDC1	6	9	5.91E-04	0.019633	0.973472
R-HSA-3656243	Defective ST3GAL3 causes MCT12 and EIEE15	6	9	5.91E-04	0.019633	0.973472
R-HSA-390522	Striated muscle contraction	16	40	0.002626	0.027115	0.973472
R-HSA-428359	Insulin-like growth factor-2 mRNA binding proteins (IGF2BPs/IMPs/VICKZs) bind RNA	7	13	8.53E-04	0.033742	0.973472
R-HSA-8864260	Transcriptional regulation by the AP-2 (TFAP2) family of transcription factors	19	52	0.003414	0.03776	0.973472
R-HSA-380108	Chemokine receptors bind chemokines	20	57	0.003742	0.047436	0.973472

Only pathways with a p-value <0.05 are shown. The entire table is found in **Supplementary Table S2**.

#### 3.1.3 Naïve skin vs. skin from the site of uninfected *I. scapularis* fed for 6 h

**Figure 4:**
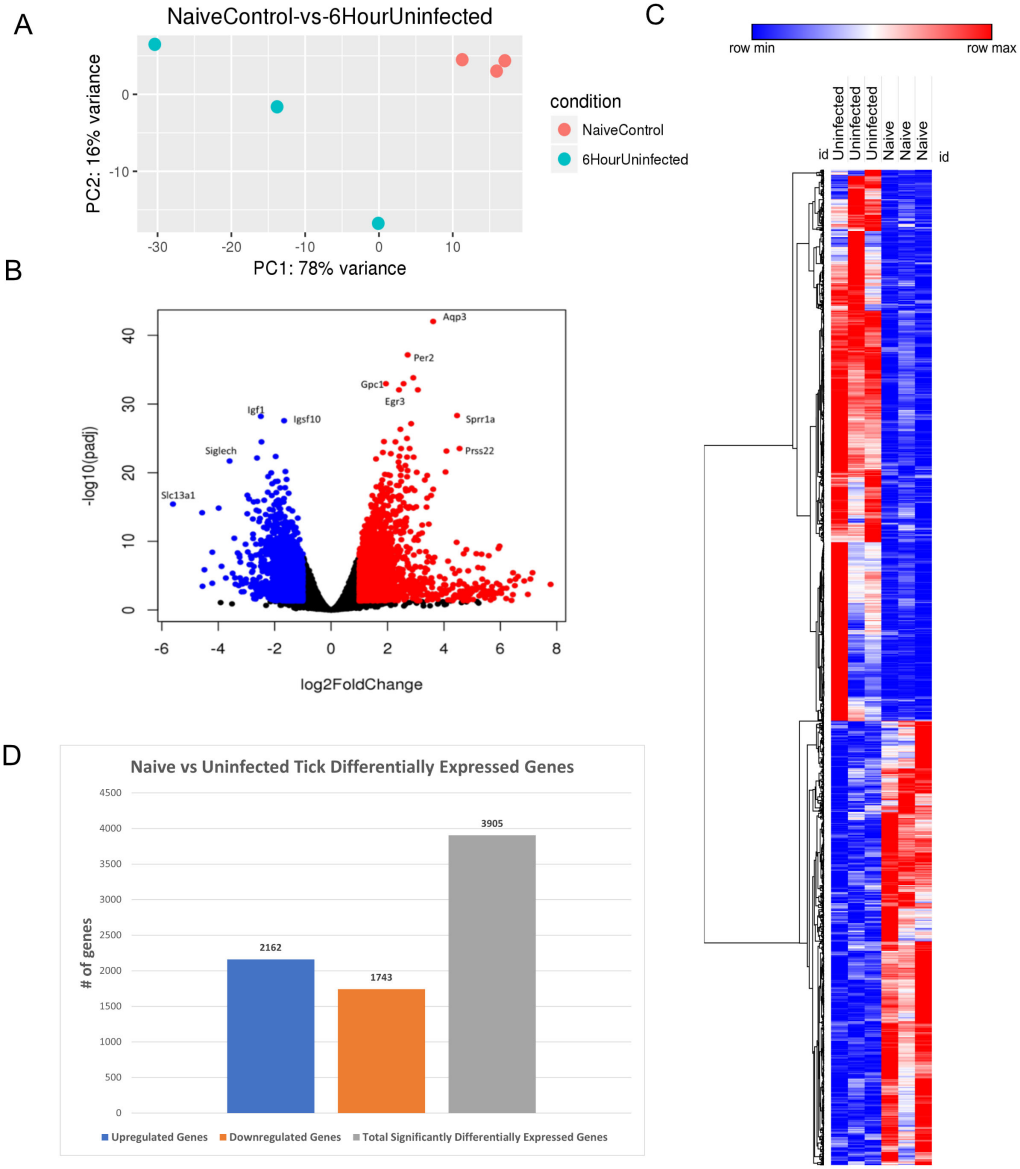
Differential expression of genes in *I. scapularis*–free control skin compared to 6-h uninfected tick feedings. **(A)** Principal component analysis (PCA) of transcriptional profiles when comparing 6-h uninfected feedings (blue) to naïve skin (orange). **(B)** Volcano plot outlining upregulated and downregulated genes at 6 h. The x-axis identifies the log2 fold change of every gene, and the y-axis identifies the log10 of its adjusted p-value. Only genes with a p-value <0.05 and a log2 fold change <−1 are identified as downregulated (blue). Only genes with a p-value <0.05 and a log2 fold change >1 are identified as upregulated (red). **(C)** A bi-clustering heatmap was used to visualize the expression profile of all differentially expressed genes (DEGs), sorted by their adjusted p-value by plotting log2-transformed values in samples. **(D)** Total DEGs for uninfected tick feeding vs. naïve skin at 6 h.

Finally, we analyzed the cutaneous transcriptomic changes during 6 h of uninfected tick feeding. PCA revealed consistent clustering of the tick-free control skin samples, with a larger variance observed in the skin samples from the 6-h uninfected tick group (**Figure 4A**).

Volcano plot analysis identified 3,905 significant DEGs, with 2,162 upregulated and 1,743 downregulated genes (**Figure 4B**). Notable downregulated genes included *Slc13a1*, *Siglech*, *Igf1*, and *Igsf10*, which participate in sodium ion transport, cell signaling, and neuron migration. Upregulated genes, such as *Aqp3*, *Per2*, *Gpc1*, *Egr3*, *Sprr1a*, and *Prss22*, were associated with cell signaling and survival. Interestingly, *Aqp3*, an aquaporin, suppresses apoptosis in skin fibroblasts by upregulating *Bcl-2*, highlighting the potential for host defense mechanisms to be activated at tick bite sites (27).

The bi-clustering heatmap allowed for comparison of all DEGs between the groups (**Figure 4C**). This summary heatmap shows the comparisons of each individual mouse for both groups, tick-free mice versus 6-h uninfected tick feeding. The tree to the left demonstrates hierarchical clustering of the samples. The differential analysis revealed a total of 3,905 genes expressed in this study (**Figure 4D**).

DEG data were input into the Reactome database, and PE analysis revealed the impacted pathways during the 6-h feeding, including keratinization, formation of the cornified envelope, and nerve growth factor (NGF)-stimulated transcription (**Table 4**).

**Table 4:** Pathway enrichment of naïve *I. scapularis*–free skin vs. skin harvested after 6 h of uninfected *I. scapularis* feedings.

Pathway identifier	Pathway name	#Entities found	#Entities total	Entities ratio	Entities p-value	Entities FDR
R-HSA-6805567	Keratinization	100	226	0.014837	1.09E-05	0.023823
R-HSA-6809371	Formation of the cornified envelope	60	138	0.00906	8.41E-04	0.920213
R-HSA-9031628	NGF-stimulated transcription	25	56	0.003676	0.018067	0.973879
R-HSA-1306955	GRB7 events in ERBB2 signaling	5	6	3.94E-04	0.028431	0.973879
R-HSA-446107	Type I hemidesmosome assembly	7	11	7.22E-04	0.037806	0.973879
R-HSA-9690406	Transcriptional regulation of testis differentiation	11	21	0.001379	0.037965	0.973879
R-HSA-8939256	RUNX1 regulates transcription of genes involved in WNT signaling	6	9	5.91E-04	0.043483	0.973879
R-HSA-8849474	PTK6 activates STAT3	5	7	4.60E-04	0.049237	0.973879

Only pathways with a p-value <0.05 are shown. The entire table is found in **Supplementary Table S3**.

### 3.2 Changes in the skin transcriptome during POWV-infected *I. scapularis* feeding

#### 3.2.1 Comparison of skin from the sites of POWV-infected vs. uninfected *I. scapularis* fed for 6 h

In this experiment, *I scapularis* females either infected with POWV or mock infected with DMEM as a control were allowed to feed on mice for 1, 3, and 6 h. After 1, 3, and 6 h of tick feeding, 4-mm skin biopsies of the bite site were excised, and DEG analysis via RNA-seq was performed as described previously. POWV transcripts were detected through ddPCR to verify infection at the time points described in this study (**Supplementary Figure S1**). Compared to the uninfected tick bites, DEG analysis performed on the 1 and 3 h post–POWV-infected tick bite sites yielded significantly fewer DEGs, prompting us to focus on the 6-h time point due to the richness of the data. Comparing POWV-infected tick feeding site with that of the uninfected tick feeding sites resulted in identifying 83 statistically significant DEGs, including 80 upregulated genes and 3 downregulated genes. The results show potentially how POWV exploits the tick’s host modulatory effects as well as identifying specific changes induced by the presence of POWV during the feeding event.

**Figure 5:**
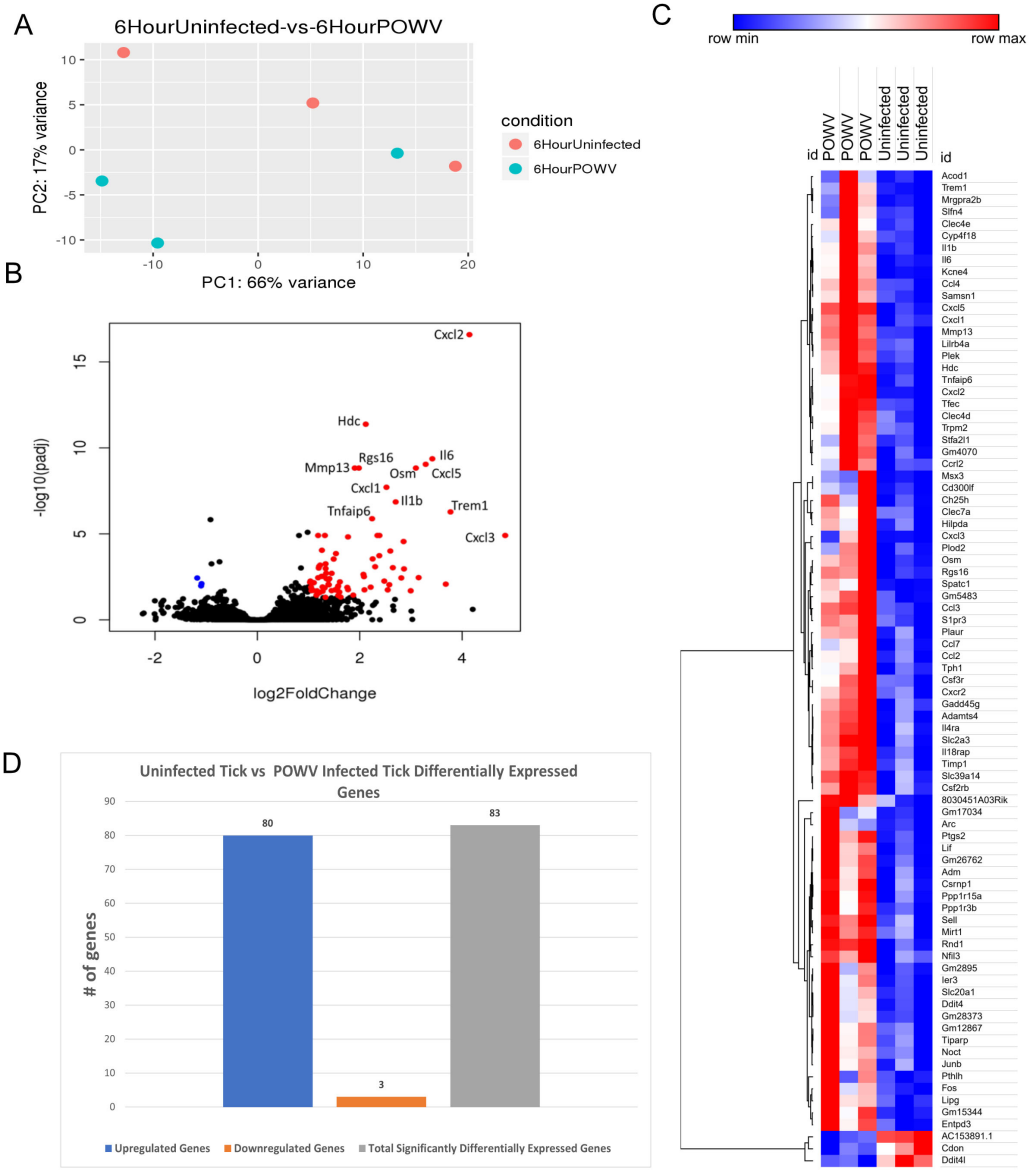
Differential gene expression at the *I. scapularis* feeding site in response to POWV-infected tick and uninfected tick bites at 6 h after attachment. **(A)** Principal component analysis (PCA) of transcriptional profiles when comparing 6-h uninfected tick feeding (blue) to 6-h POWV-infected tick feeding (orange). **(B)** Volcano plot outlining upregulated and downregulated genes at 6 h. The x-axis identifies the log2 fold change of every gene, and the y-axis identifies the log10 of its adjusted p-value. Only genes with a p-value <0.05 and a log2 fold change <−1 are identified as downregulated (blue). Only genes with a p-value <0.05 and a log2 fold change >1 are identified as upregulated (red). **(C)** A bi-clustering heatmap was used to visualize the expression profile of top 30 differentially expressed genes (DEGs), sorted by their adjusted p-value by plotting log2-transformed values in samples. **(D)** Total DEGs for uninfected tick feeding vs. POWV-infected tick feeding at 6 h.

PCA for the 6-h uninfected tick feeding sites versus the POWV-infected tick-feeding sites revealed variance between uninfected samples, with one observed outlier among the POWV-infected samples (**Figure 5A**).

Gene expression analysis using volcano plots highlighted a substantial upregulation of immune regulatory genes after 6 h of POWV-infected tick feeding (**Figure 5B**). Prominent among the upregulated genes were those associated with neutrophil recruitment, including *Cxcl3*, *Cxcl2*, *Mrgpra2b*, *Il-6*, *Cxcl5*, *Il-1b*, and *Cxcl1* (28, 29). Additionally, genes involved in macrophage and natural killer cell migration, such as *Ccl4* and *Ccl3*, were also upregulated (30, 31). Furthermore, a broad cell surface receptor, Trem1, was highly upregulated, suggesting its role in enhancing immune responses (32). Notably, the only significantly downregulated genes during the 6-h feeding were *AC153891.1*, *Ddit4l*, and *Cdon*, with the latter being a cell adhesion molecule involved in myogenic differentiation (33, 34).

Heatmap analysis of all DEGs at this time point demonstrated clustering within the groups. Despite the smaller number of DEGs, this clustering enables the visualization of individual changes in each sample. Notably, the three downregulated genes, *AC153891.1*, *CDON*, and *DDIT4L*, clustered together (**Figure 5C**). The differential analysis revealed a total of 83 genes expressed in this study (**Figure 5D**).

**Figure 6:**
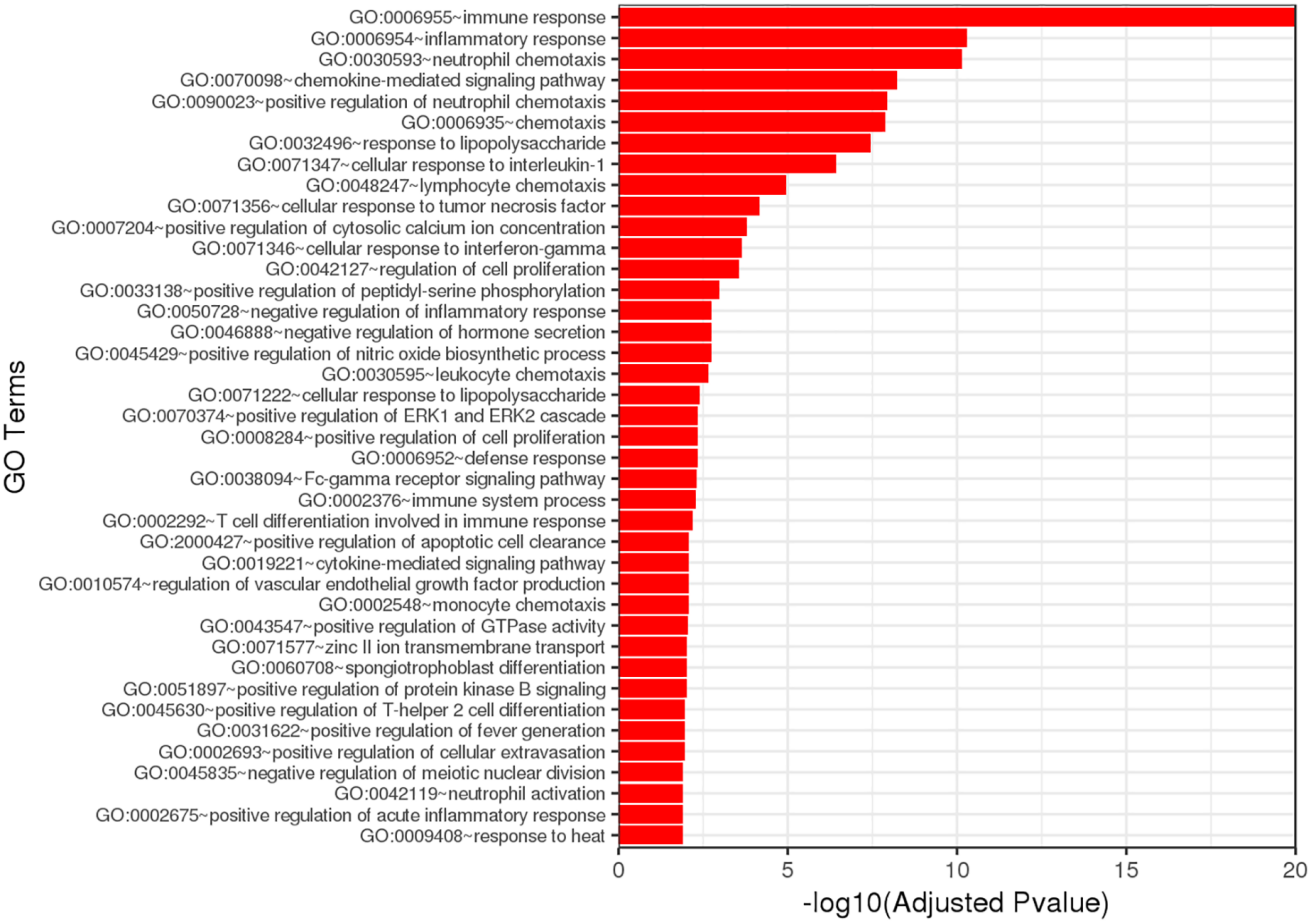
Gene ontology (GO) analysis based on DEG analysis shows strong signaling effects on neutrophil chemotaxis and inflammation.

GO enrichment analysis of the DEGs identified significant enrichment of pathways related to neutrophil chemotaxis, chemokine-mediated signaling, and the inflammatory response (**Figure 6**). Reactome pathway enrichment analysis identified several immune-related pathways impacted by the DEGs, including Interleukin (IL)-4 and IL-13 signaling, IL-10 signaling, cytokine signaling, and immune system functions (**Table 5**).

**Table 5:** Pathway enrichment of 6-h uninfected *I. scapularis* feedings vs. 6-h POWV-infected *I. scapularis* feeding.

Pathway identifier	Pathway name	#Entities found	#Entities total	Entities ratio	Entities p-value	Entities FDR
R-HSA-6785807	Interleukin-4 and interleukin-13 signaling	22	211	0.013852	1.11E-16	9.88E-15
R-HSA-6783783	Interleukin-10 signaling	21	86	0.005646	1.11E-16	9.88E-15
R-HSA-449147	Signaling by interleukins	33	658	0.043199	1.11E-16	9.88E-15
R-HSA-380108	Chemokine receptors bind chemokines	13	57	0.003742	1.11E-15	7.33E-14
R-HSA-1280215	Cytokine signaling in immune system	33	1039	0.068212	1.13E-12	5.96E-11
R-HSA-168256	Immune system	48	2627	0.172466	9.11E-10	4.01E-08
R-HSA-375276	Peptide ligand–binding receptors	13	203	0.013327	6.41E-09	2.44E-07
R-HSA-373076	Class A/1 (rhodopsin-like receptors)	14	414	0.02718	3.60E-06	1.19E-04
R-HSA-9031628	NGF-stimulated transcription	6	56	0.003676	5.18E-06	1.50E-04
R-HSA-500792	GPCR ligand binding	16	609	0.039982	1.65E-05	4.29E-04

Only the top 10 pathways with a p-value <0.05 are shown, due to table size. The entire table is found in **Supplementary Table S4**.

**Figure 7:**
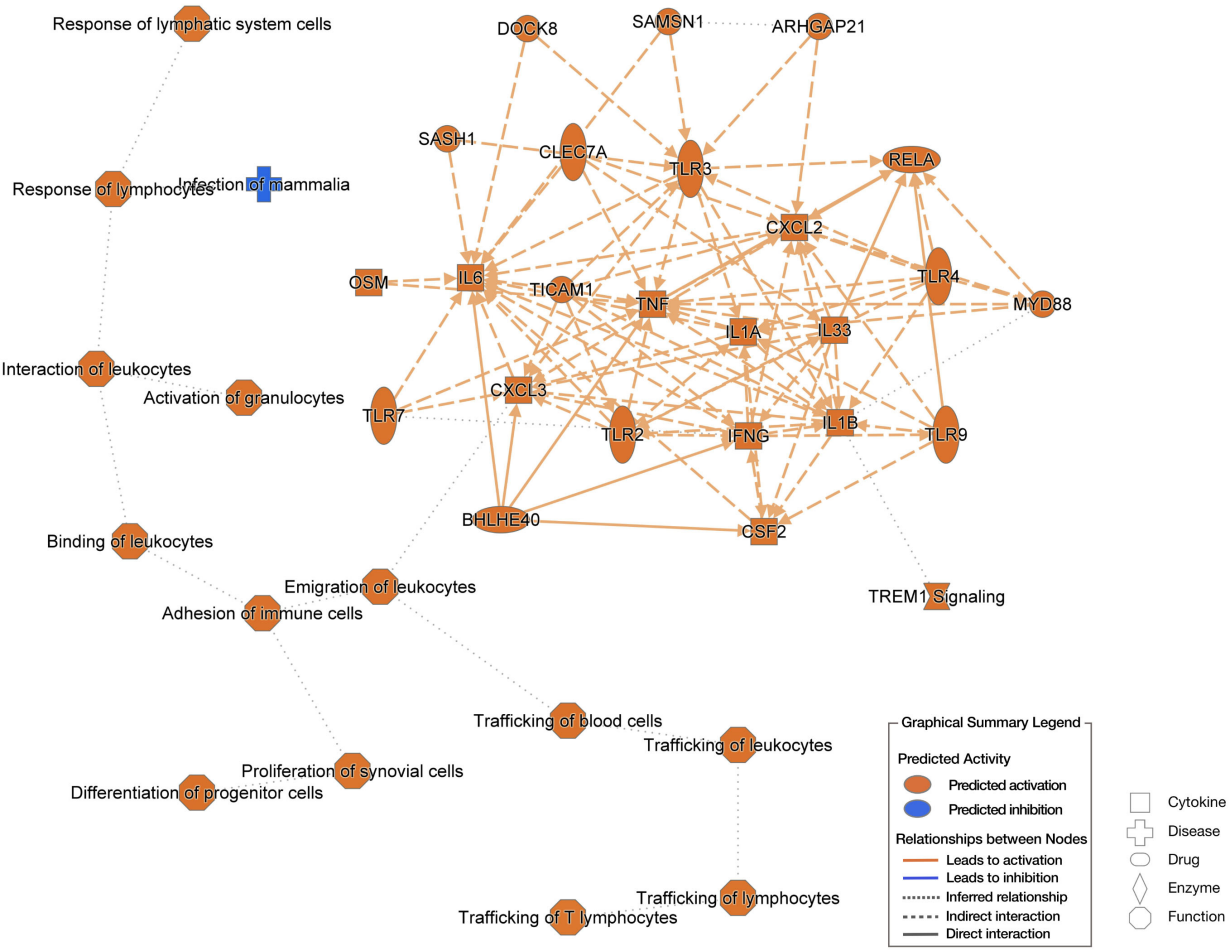
Ingenuity pathway analysis (IPA) based on 6-h uninfected *I. scapularis* feedings vs. 6-h POWV-infected feedings. The left side of the image shows extracellular predictions based on input information, and the right-side network represents potential molecular interactors based on input information. Predictions made using Qiagen IPA algorithms.

To gain a comprehensive understanding of the cutaneous transcriptomic changes during the 6-h POWV feeding, we utilized ingenuity pathway analysis (IPA). The network interaction summary (**Figure 7**) depicts the interaction between downstream and upstream pathways during the initial stages of POWV infection, specifically at the 6-h time point after tick attachment. Here, we can see the changes that are a result of POWV infection, where the left side of the chart shows the extracellular components that may be upregulated or activated on the basis of infection status. These include things that we would expect to be stimulated such as recruitment of all different cell types like lymphocytes, leukocytes, blood cells, and progenitor cells. The matrix on the right highlights chemokines such as CXCL3 that participate in the recruitment of leukocytes to the bite site, as well as the trafficking of other immune cells and signaling components to the affected area. Most of the upregulated genes identified in this study are associated with inflammation and activation of an immune response, especially early innate responses. Components leading to major signaling like v-rel avian reticuloendotheliosis viral oncogene homolog A (RELA), chemokine (C-X-C motif) ligand 2 (CXCL2), myeloid differentiation primary response 88 (MYD88), and tumor necrosis factor (TNF) are implicated. These findings align with previous work, suggesting that immune cell infiltration and inflammatory responses are important drivers of the host’s reaction to initial POWV infection transmitted by ticks (19, 23, 35).

## 4 Discussion

The initial hours and days of tick feeding has been linked to the transmission of a wide variety of pathogens, including viruses, bacteria, and parasites, each with a specific window of time required for transmission (15, 16, 36–41). As shown in **Table 1** where DEGs for each bioinformatic analysis were outlined, minimal changes were observed at the 1-h time point when comparing POWV-infected and uninfected tick feeding sites. This could primarily be due to the initial tick attachment, during which there may not be sufficient time for the virus or tick salivary factors to significantly alter the attachment site. It is possible that 1 h of POWV-infected tick feeding is insufficient to elicit robust transcriptional changes in the skin. However, the detection of 3,722 significant DEGs resulting from uninfected tick feedings, compared to an unbitten mouse, indicates immunomodulatory changes at the tick feeding site. This is supportive of our current understanding of how arthropod feedings cause an enumerative number of local changes at their feeding site. The primary alterations involved genes associated with coagulation and wound healing factors, likely due to the introduction of tick salivary components, as identified in the bi-clustered heatmap (**Figure 2**). For example, during the initial feeding stages, tick saliva contains various anti-hemostatic factors that prevent coagulation and enhance blood meal acquisition (42).

At 6 h after tick attachment during POWV-infected tick feedings versus uninfected tick feedings, we observed the upregulation of proinflammatory genes and those involved in immune cell recruitment. This is consistent with previous findings on the early recruitment of macrophages and neutrophils, as demonstrated through PCR arrays and histological examination of the POWV-infected *I. scapularis* feeding site (17, 18). The upregulation of several transcripts associated with neutrophil recruitment, such as Cxcl3, Cxcl2, Mrgpra2b, Il-6, Cxcl5, Il-1b, and Cxcl1, supported by pathway enrichment and GO analysis, indicates the establishment of a strong proinflammatory environment at the POWV-infected tick feeding site. Neutrophils are known to be early responders to viral infections, recognizing pathogen-associated molecular patterns (PAMPs) and being stimulated by chemokines. For example, during influenza infection, virally infected cells secrete Cxcl8 and granulocyte macrophage-colony stimulating factor to attract neutrophils (43). Neutrophil activation is also observed in other arbovirus infections. Zika virus, for instance, works in concert with *Aedes aegypti* salivary protein neutrophil-stimulating factor 1 (NeSt1) to promote neutrophil recruitment, enhancing Zika virus pathogenesis in mice (44). Our data supports previous findings regarding the early influx of neutrophils to the site of POWV-infected nymphal tick feeding (17, 18). The important distinction here is the use of adult *I. scapularis* ticks, which are known to have different salivary composition, feeding behavior, and infection kinetics for POWV (10, 45–47). The influx of neutrophils to sites of infection or tissue damage is not novel; however, their specific role in the pathogenesis of POWV transmission and dissemination is unknown. As our DEG analysis yields strong correlation between POWV, neutrophil recruitment, and inflammation, we can potentially surmise a key role these cells have during initial POWV infection. For *Borrelia* sp. infections, neutrophil recruitment and degranulation play critical roles in limiting the infection through extracellular traps, phagocytosis, and oxidative bursts (48). The role of these factors in determining disease outcomes during POWV infections remains unclear. However, even uninfected tick feeding induces the recruitment of neutrophils and other immune cells to the feeding site. This effect is amplified in the presence of POWV, with infected fibroblasts further contributing to the inflammatory response by increasing cytokine release upon infection.

Interestingly, in terms of inflammation and early innate activation, we do not observe many genes associated with interferon stimulation and activation. From our data, it is clear that *SOCS3* (suppressor of cytokine signaling 3) and *IFITM1* (interferon-induced transmembrane protein 1) as the primary upregulated components involved in the interferon pathway. SOCS3 is a well-described anti-interferon component of the immune response, used primarily to subdue the inflammatory response when not needed (49). This is in contrast to IFITM1, which is strongly upregulated by type I and II interferons to play a role in antiproliferation in context of viral infections (50). These two may be working in opposite directions, depending on what is interacting with them. Investigations into POWV and the interferon response are areas that should be prioritized by further research.

The generation of a proinflammatory state is a critical aspect of disease pathogenesis, particularly during viral transmission and dissemination. At the 6-h post–POWV-infected tick feeding, we observed a broad array of inflammatory markers upregulated through IPA. These markers included various canonical inflammation pathways associated with viral responses, notably *IL-1b, IL-6*, and *CCL2*, each showing a greater than 2-log expression increase. These markers suggest the involvement of the NLRP3 inflammasome pathway during POWV transmission. The NLRP3 inflammasome is a well-established marker for inflammation during pathogen infiltration, regulated by both PAMPs and damage-associated molecular patterns (51). The activation of the NLRP3 inflammasome has been described for many arboviruses, particularly those spread by mosquitoes. For example, the dengue virus activates NLRP3 by using NS2A and NS2B proteins to initiate an inflammatory environment, leading to the recruitment of permissive cells (52). Similarly, markers such as IL-1b and IL-6 are recognized biomarkers for Chikungunya disease severity in clinical settings (53, 54). Although these components are heavily induced during POWV transmission, their roles in tick-borne diseases are not well characterized. A closely related virus, tick-borne encephalitis virus (TBEV), is a neuroinvasive tick-borne virus prevalent in Europe and Asia. A cohort study identified TNFα, IL-1b, and IL-6 as being highly prevalent in the serum of patients upon hospital admission, suggesting that these cytokines play a crucial role in TBEV infection and pathogenesis (55). NLRP3 is a well-established inflammatory holoenzyme that activates in response to many arboviruses (52, 56–58). The potential involvement of the NLRP3 inflammasome in POWV pathogenesis warrants further investigation into the roles of these immune responses. Whereas the primary products of this pathway were identified to be significantly upregulated, *NLRP3* transcript was found to be upregulated but not statistically significant in response to POWV-infected tick bites at 6 h after tick attachment. This is attributed to that NLRP3 being constitutively expressed even in response to uninfected tick bites. Assessing its role in the future is critical for understanding pathogenesis and immune interaction with POWV.

**Figure 8:**
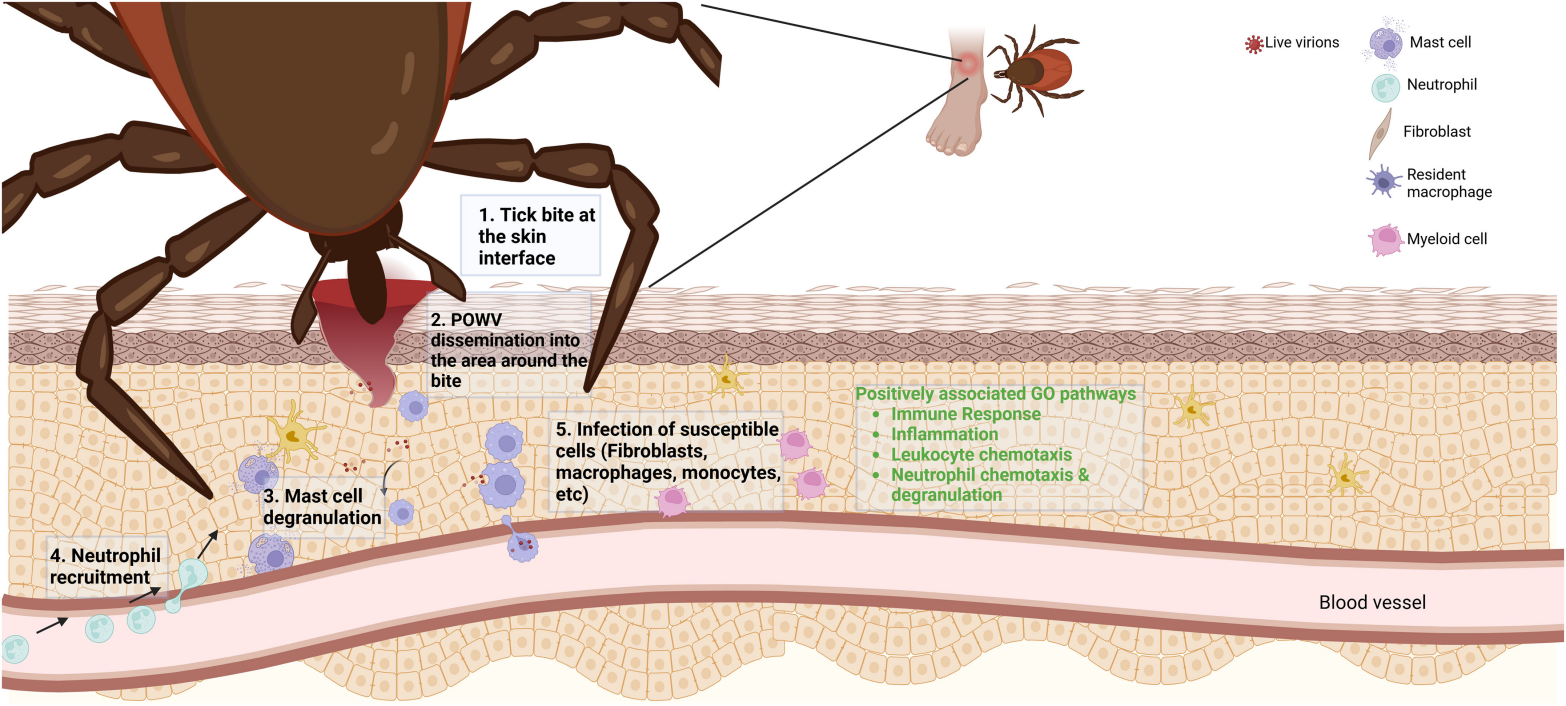
A proposed Powassan virus (POWV) transmission model based on differentially expressed genes. Upon transmission of POWV via an infected *I. scapularis*, the virus infects local cells at the bite site, resulting in differential expression of specific genes. The expression of various transcripts may yield recruitment of lymphocytes to the area of infection. The recruitment of these cells to the area of infection could result in them becoming infected, potentially allowing for the establishment of infection. In green text are highly implicated pathways of involvement from GO analysis of 6-h tick feedings with POWV.

During POWV transmission from tick to vertebrate host, the virus encounters a variety of resident and recruited immune cells at the tick feeding site. Resident macrophages and Langerhans cells, which commonly encounter pathogens introduced into the skin, have been shown to co-localize with POWV RNA in previous studies using RNA *in situ* hybridization (35). This previous study was conducted 24 h after attachment with *I. scapularis* nymphs and aimed to address what occurs at the critical time points relevant to transmission. The increased analyte count from RNA-seq in our current study allows for a more detailed investigation, reinforcing the establishment of an inflammatory environment at the bite site during POWV transmission. Our proposed model for POWV infection suggests that, upon successful attachment and feeding by a POWV-infected tick, infectious virions and tick saliva are secreted into the bite site resulting in the recruitment of lymphocytes such as neutrophils. Recruitment of these cells occurs through chemokines identified in our RNA-seq analysis such as *CCL2*. There are also potential interactions with resident mast cells indicated by this study’s transcriptomic identification of *CPA3* and other cell surface ligands such as *TLR 3, 7, 8*, and *9* for pathogen detection. Resident immune cell activation and recruitment result in a positive feedback loop of chemokines and cytokines, drawing more susceptible cells to the site of infection and allowing the virus a potential route for dissemination and infection establishment (**Figure 8**).

## 5 Conclusion

Our findings suggest that POWV-infected tick feeding induces the expression of proinflammatory and immune response genes at the tick feeding site, which likely contributes to the recruitment of neutrophils and other immune cells. POWV appears to enhance the tick’s natural modulation of the host’s cutaneous cellular response to wounding via tick feeding, amplifying inflammatory pathways that may contribute to the pathogenesis of the infection. The role of neutrophils has not been heavily investigated in response to POWV infection but opens a potential area, which may lead to further studies involving the viral dissemination from skin to CNS.

## Data Availability

The data presented in the study are deposited in to the Gene Expression Omnibus, accession number GSE282786.

## Supplementary material

Supplementary Figure 1 POWV infection was assessed at the skin interface during appropriate times of an *I. scapularis* feeding. To verify the infection at each of the bite sites, ddPCR was used for each sample used in the bioinformatic analysis study.
