# Supplementary figures and images for: Early transcriptomic changes at the skin interface during Powassan virus transmission by Ixodes scapularis ticks
Source: Front Immunol. 2025 Jan 13;15:1511132. doi: 10.3389/fimmu.2024.1511132 (PMC11769802; doi:10.3389/fimmu.2024.1511132)

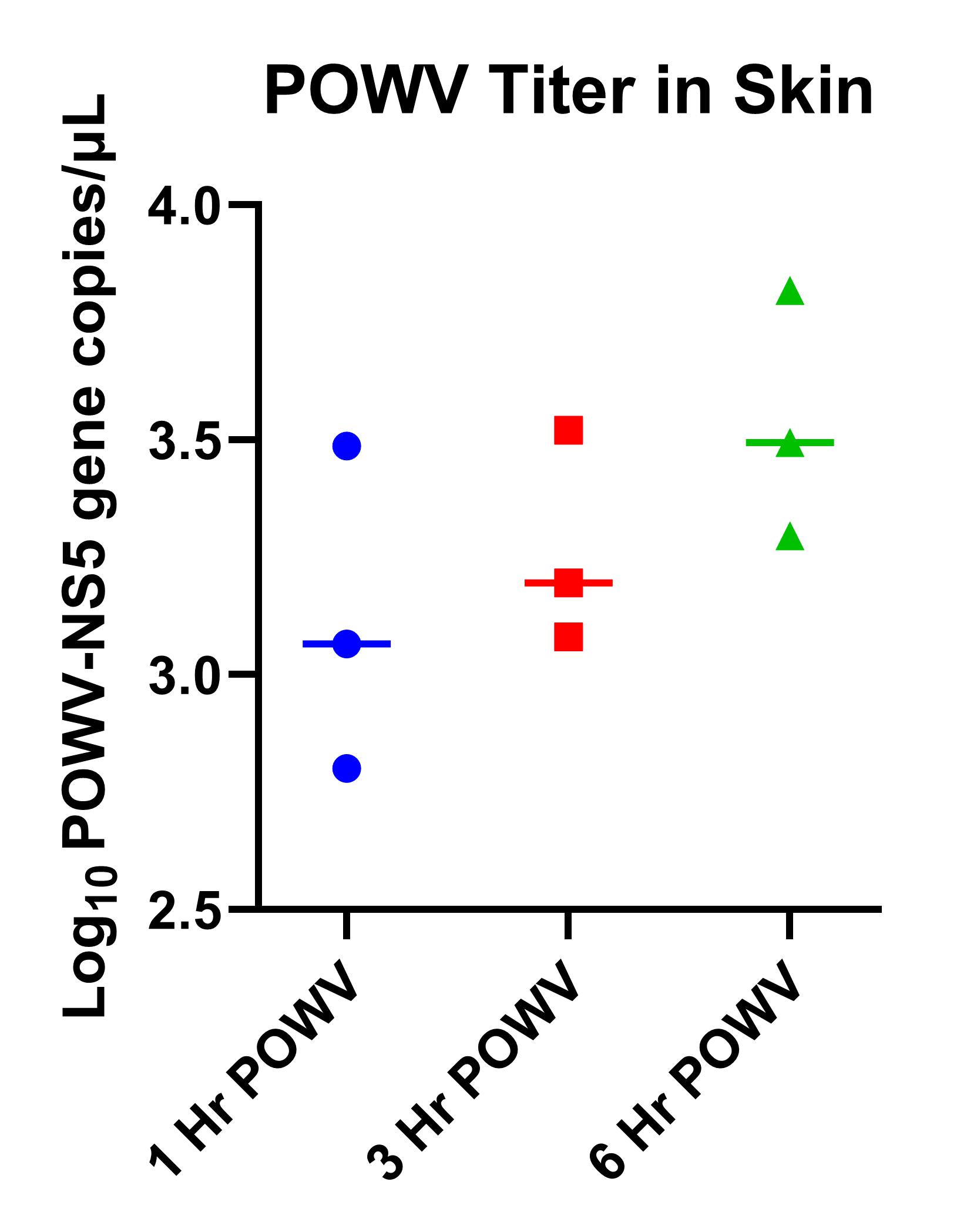

Supplement: Supplementary Figure 1 — POWV infection was assessed at the skin interface during appropriate times of an I. scapularis feeding. To verify the infection at each of the bite sites, ddPCR was used for each sample used in the bioinformatic analysis study. [file Image1.jpeg]
